# Persistencia de síntomas post COVID-19 a los dos años de la infección: Seguimiento de una cohorte en Atención Primaria

**DOI:** 10.23938/ASSN.1101

**Published:** 2025-03-12

**Authors:** Yolanda Barrera Martínez, Gerardo Andrés Boillat Oriani, Pedro Vega Montes, Elena Martínez Moreno, Alejandro Pérez Pérez, Ricardo José Casajuana Pérez, Francisca Muñoz Cobos

**Affiliations:** 1 Servicio Andaluz de Salud Atención Primaria de Salud Centro de Salud El Palo Málaga España; 2 Servicio Andaluz de Salud Atención Primaria de Salud Centro de Salud Rincón de la Victoria Rincón de la Victoria Málaga España

**Keywords:** Síndrome Post Agudo de COVID-19, Calidad de Vida Relacionada con la Salud, Atención Primaria, Estudios de Cohortes, Post-Acute COVID-19 Syndrome, Health-related quality of life, Primary Care, Cohort Studies

## Abstract

**Fundamento:**

Determinar la prevalencia de COVID persistente en los dos años tras la primo-infección, identificar sus factores pronóstico y evaluar su impacto en la calidad de vida.

**Metodología:**

Estudio ambispectivo de cohortes realizado en pacientes ≥18 años adscritos a dos centros de salud (Málaga, España), con PCR/test de antígeno positivo para SARS-CoV-2 entre octubre/2020 y mayo/2021. Se realizó un muestreo aleatorio sistemático en octubre de 2022, asumiendo precisión=5%, confianza=95% y pérdidas=25%. El seguimiento finalizó en mayo/2023. Variables dependientes: COVID persistente (≥1 síntoma durante ≥8 semanas), número de síntomas, calidad de vida (EuroQol 5-D) y percepción global de salud (EQ-EVA). Variables independientes: edad, sexo, gravedad de la infección inicial, vacunación, comorbilidades, reinfección.

**Resultados:**

Se muestrearon 173 pacientes de 914 elegibles, con edad media 47 años y 58,4% mujeres. El 32,36% presentaron COVID persistente en algún momento y un 23% a los dos años de la primoinfección, siendo los síntomas más frecuentes astenia y anosmia/disgeusia. Los factores pronóstico de COVID persistente fueron primoinfección más grave, menor edad y reinfección, mientras depresión, primoinfección más grave y reinfección lo fueron para mayor número de síntomas. La percepción global de salud fue ocho puntos menor en pacientes con COVID persistente (77,72; DE=17,10 vs 86,15; DE=16,25; p <0,001). Sexo femenino, mayor edad, menos comorbilidades y más dosis vacunales predijeron mayor calidad de vida.

**Conclusiones:**

El 32% de pacientes presentó COVID persistente, principalmente astenia y anosmia/disgeusia. La persistencia se asoció con mayor gravedad inicial, menor edad y reinfección, afectando negativamente la calidad de vida.

## Abstract (en)

**Background:**

This study aims to determine the prevalence of long COVID two years after initial infection, identify prognostic factors, and assess its impact on quality of life.

**Methodology:**

An ambispective cohort study was conducted with patients aged ≥18 years from two health centers in Málaga, Spain, who tested positive for SARS-CoV-2 between October 2020 and May 2021. Systematic random sampling was performed in October 2022, with 5% precision, 5% alpha error, and 25% expected losses. The cohort was followed until May 2023. Dependent variables included long COVID (≥1 symptom lasting ≥8 weeks), symptom count, quality of life (EuroQol 5-D), and global health perception (EQ-EVA). Independent variables included age, sex, severity of initial infection severity, vaccination status, comorbidities, and reinfection.

**Results:**

Of 914 eligible individuals, 173 patients were sampled (mean age 47 years, 58.4% female). Long COVID was observed in 32.36% of participants, with 23% affected two years after infection. Fatigue and anosmia/dysgeusia were the most frequent symptoms. Prognostic factors for long COVID included higher initial infection severity, younger age, and reinfection. For symptom count, factors were depression, initial infection severity, and reinfection. Health perception was eight points lower in long COVID patients (77.72: SD=17.10 vs 86.15; SD=16.25; p<0.001). Female sex, older age, fewer comorbidities, and more vaccine doses were associated with better quality of life.

**Conclusions:**

Thirty-two percent of patients experienced long COVID, mainly fatigue and anosmia/dysgeusia. It associates with higher initial severity, younger age, and reinfection, negatively impacting quality of life.

## INTRODUCCIÓN

La infección por coronavirus del síndrome respiratorio agudo grave de tipo 2 (SARS-CoV-2) ha supuesto una emergencia sanitaria internacional^1^^,^^2^^,^^3^. Con la evolución de la pandemia han aparecido nuevos focos de atención como la COVID persistente o *long* COVID^4^^,^^5^, término para el que aún no existe una descripción única^4^^,^^6^ y que la Organización Mundial de la Salud (OMS) define como la condición que ocurre en personas con antecedentes de infección por SARS-CoV-2 que presentan síntomas variados, con duración mínima de ocho semanas y que afectan al funcionamiento diario, sin que puedan explicarse por diagnósticos alternativos^7^.

Debido a la disparidad de definiciones, la prevalencia publicada de COVID persistente es variable y puede afectar al 15% de las personas con infecciones sintomáticas y al 6,8% de todas las infecciones^8^. Respecto a la duración, un estudio realizado en España, se encontró una media de 185 días de persistencia de los síntomas^9^. La lista de manifestaciones clínicas es amplia y heterogénea^10^^,^^11^, con más de 200 síntomas descritos^9^^,^^11^. Un metaanálisis estimó una prevalencia del 80%, incluyendo pacientes con uno o más síntomas, dos semanas tras la infección aguda; durante el seguimiento (entre 14 y 110 días) se identificaron 55 síntomas a largo plazo, siendo los más frecuentes fatiga (58%), cefalea (44%), trastorno de la atención (27%), pérdida de cabello (25%) y disnea (24%)^12^. Otro metaanálisis posterior encontró una prevalencia menor (43%) en los 28 días posteriores a la infección, siendo mayor en pacientes hospitalizados frente a no hospitalizados (54 vs 34%); el síntoma más frecuente fue la fatiga (23%), seguido de fallos de memoria (14%) y disnea (13%)^13^. En un estudio con datos agregados por grupos de síntomas, un 98,3% de pacientes afectados de COVID persistente presentaban síntomas generales, 93,9% del aparato locomotor, 93% cardiorrespiratorios, 88,3% psicológicos y emocionales, 88% neurológicos y 85,5% digestivos, mostrando así afectación multisistémica^11^. En una encuesta realizada en España a pacientes con COVID persistente se recogieron 200 síntomas, encontrando una media de 36 por persona, con gran impacto sobre la calidad de vida y funcionalidad^14^. En un estudio multicéntrico, el 63,9% de pacientes hospitalizados en la fase aguda de COVID-19 presentaba síntomas a los seis meses, siendo los respiratorios (42%) y sistémicos (36,1%) los más frecuentes, seguidos por los neurológicos (20,8%) y psicológicos (12,2%)^15^.

La fisiopatología de la COVID persistente parece depender tanto de mecanismos patogénicos del virus como de la respuesta fisiológica del paciente^14^. Se han propuesto distintos mecanismos explicativos del daño, como hiperinflamación crónica, ruptura de la integridad de la barrera hematoencefálica, estado de hipercoagulabilidad, alteración del sistema nervioso autónomo y afectación inmunológica^16^^,^^17^. No conocemos claramente por qué ciertos síntomas se hacen persistentes en algunas personas infectadas^18^.

El transcurso de la COVID persistente es variable, existiendo casos sin mejoría durante el seguimiento del paciente, casos fluctuantes con intervalos de exacerbación y remisión de los síntomas, y casos con una mejoría lentamente progresiva^18^^,^^19^. Se han sugerido factores de riesgo para desarrollar COVID persistente^20^, como sexo femenino, edad elevada, mayor número de síntomas (≥5 síntomas en la primoinfección)^4^ y su tipo (fatiga, cefalea, disnea)^21^, sin una relación clara con la gravedad de la infección inicial. Además, la anosmia se ha identificado como un síntoma predictivo clave para el desarrollo de COVID persistente^14^^,^^22^.

Respecto al impacto personal, se ha descrito la necesidad de mayor asistencia médica posterior^23^, lenta reincorporación laboral (solo del 40% en los 2-3 meses tras la infección aguda)^24^, aumento de reingresos y de mortalidad^25^. Los pacientes han declarado un empeoramiento de su salud^14^, con un descenso medio de 6,32 puntos en una escala de 0 a 10, y una repercusión negativa en la calidad de vida(medida mediante la escala visual analógica EQ-EVA del instrumento *European Quality of Life-5 Dimensions* - EuroQol-5D) con una puntuación media de 75,8 en una escala de 0 a 100 y desviación estándar 18,7^26^; la calidad de vida fue significativamente menor en mujeres que hombres, mayores de 65 años respecto a los menores, pacientes con comorbilidades frente a los que no las presentan y con necesidad de ingreso hospitalario respecto a los que no lo requirieron.

La atención primaria es el nivel asistencial cuyos pacientes se acercan más a la situación de salud y enfermedad poblacional, atendiendo a la mayoría de pacientes con COVID (infecciones leves-moderadas). Sus características (visión global e integral del paciente, modelo biopsicosocial, atención longitudinal) son fundamentales en la atención a patologías multisistémicas que afectan a la calidad de vida y se manifiestan a lo largo del tiempo, como es la COVID persistente^27^, si bien es necesario aportar más conocimientos sobre esta patología seguida en el primer nivel asistencial y extender la evaluación de su comportamiento clínico durante un tiempo prolongado.

Este estudio pretende conocer la prevalencia de síntomas de COVID persistente a largo plazo (dos años de seguimiento desde la primoinfección) en pacientes de atención primaria, evaluando los síntomas más frecuentes, la repercusión en la calidad de vida y las posibles diferencias por factores clínicos y sociodemográficos.

## MATERIAL Y MÉTODOS

Estudio de cohortes realizado en pacientes con COVID-19 confirmada dos años antes de la recogida de datos entre octubre 2022 y mayo de 2023 en Atención Primaria de Málaga (España).

Se realizó en dos centros de salud. El Centro de Salud El Palo atiende a una población de 44.500 habitantes e incluye dos consultorios rurales, uno correspondiente a un núcleo semi-rural (Olías) y otro a un municipio de 710 habitantes (Totalán). La Zona Básica de Salud del Rincón de la Victoria atiende a una población de 47.000 habitantes; incluye el Centro de Salud del Rincón de la Victoria y siete consultorios rurales: La Cala del Moral, Benagalbón, Torre de Benagalbón, Moclinejo, Valdéz, Macharaviaya y Benaque.

Los criterios de inclusión de pacientes fueron: 1) ser mayor de edad (≥18 años), 2) adscrito a uno de los dos centros de salud, 3) con infección por SARS-CoV-2 confirmada mediante PCR o test de antígeno entre el 1 de octubre de 2020 y el 31 de mayo de 2021 y, 4) aceptar su participación mediante la firma del consentimiento informado.

Se realizó el cálculo del tamaño muestral necesario para estimar la prevalencia de síntomas a los 24 meses con un 95% de nivel de confianza (z=1,96) y un 5% de precisión (d=0,05). Los estudios de COVID persistente realizados muestran resultados de prevalencia dispares y heterogéneos (20-80%), dependiendo de la definición considerada y los criterios de inclusión y del tiempo de seguimiento. Para el cálculo del tamaño muestral se consideró una prevalencia esperada de COVID persistente del 10% (p=0,10), dado que en dos años tras la primoinfección la persistencia de síntomas podría disminuir. 

Con estos parámetros, y mediante la fórmula $n=\frac{Z^{2}\;pq}{d^{2}}$ , se requiere una muestra de 139 pacientes. En previsión de un 25% de posibles pérdidas debido al largo tiempo desde la infección a la recogida de datos, la muestra se aumentó a 172 personas.

**Figura 1. Proceso:**
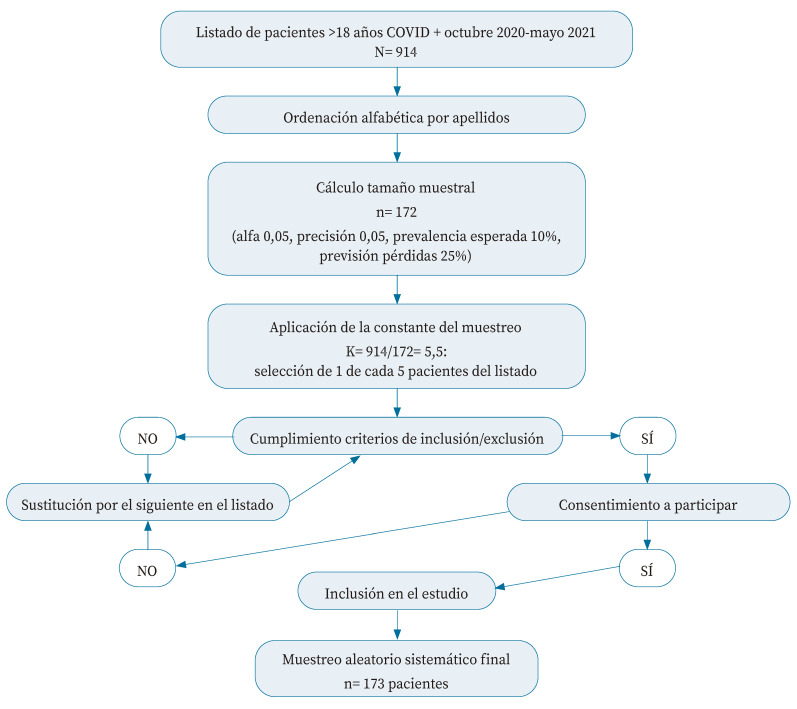
de selección de la muestra.

Al aplicar K=5 como constante de muestreo en lugar de la obtenida del cálculo matemático (K=914/172=5,3) se seleccionó una persona más al número necesario para cumplimentar el tamaño muestral. Por tanto, se seleccionó aleatoriamente una de cada cinco personas participantes a partir del listado de 914 pacientes con diagnóstico COVID en las fechas y por los métodos señalados, numeradas según orden alfabético de apellidos. Si el caso seleccionado no cumplía criterios de inclusión era sustituido por el siguiente de la lista (Fig. 1). Todos los pacientes incluidos dieron su consentimiento a participar en el estudio. La investigación contó con el permiso del Comité Provincial de Ética de la Investigación de Málaga emitido con fecha 1-3-2022.

Los datos se obtuvieron de las historias clínicas informatizadas de pacientes y por entrevista clínica presencial o telefónica entre octubre 2022 a mayo de 2023, respetando en la recogida de variables el marco temporal de 24 meses post primoinfección por SARS-CoV-2.

Se consideraron tres variables dependientes:

- Persistencia de síntomas COVID, definida como la presencia de al menos un síntoma durante un periodo ≥8 semanas sin otra causa atribuible desde la infección por SARS-CoV-2 en algún momento de los dos años postinfección. El listado de síntomas se extrajo de la literatura consultada^11^^,^^12^: febrícula, escalofríos, astenia, malestar general, artralgias y mialgias, disnea, tos seca, opresión torácica, palpitaciones, ansiedad, apatía, trastornos del sueño, cefalea, parestesias, anosmia, disgeusia, pérdida de audición, déficit de memoria, inestabilidad, mareo, incapacidad para concentrarse, dolor abdominal, dispepsia, diarrea, vómitos.
- Número de síntomas persistentes.
- Calidad de vida autopercibida relacionada con la salud, medida mediante el cuestionario EuroQol 5-D que evalúa cinco dimensiones (movilidad, cuidado personal, actividad cotidiana, dolor y ansiedad/depresión), y mediante una escala visual análogica (EQ-EVA) que evalúa la percepción global de la salud de 0 (peor estado posible) a 100 (mejor estado posible)^17^.

También se recogieron las siguientes variables independientes:

- demográficas: sexo (hombre, mujer), edad (años cumplidos);
- clínicas: tipo de comorbilidades (depresión, obesidad, hipertensión arterial, diabetes, enfermedad pulmonar obstructiva crónica/EPOC, cardiopatía, accidente cerebrovascular, insuficiencia renal, cáncer), número de comorbilidades, embarazo, inmunosupresión;
- relacionadas con la COVID-19: vacunación previa (sí, no y número de dosis), gravedad de los síntomas (síntomas ausentes/escasos, leves que no requirieron ingreso hospitalario, graves que requirieron ingreso hospitalario en planta o en unidad de cuidados intensivos/UCI; se dicotomizó en asintomáticos/sintomáticos), reinfección por SARS-CoV-2.

### Análisis estadístico

Las variables cuantitativas se describieron mediante media y desviación estándar (DE) y se compararon con t-Student o, si no se cumplen sus requisitos de aplicación, la prueba no paramétrica U de Mann-Withney. Las variables se describieron mediante frecuencia y porcentaje, y se compararon con Chi-cuadrado. Se realizó análisis multivariante de regresión lineal para las dos variables dependientes cuantitativas, y de regresión logística para la variable dependiente dicotómica, por el método paso a paso. El nivel de significación fue 95%. Se utilizó el programa estadístico SPSS versión 23.0.

## RESULTADOS

Se incluyeron 173 pacientes, con una edad media de 47 años (rango 19-89) y predominio de mujeres (58,4%). La mitad de la muestra (50,9%) presentaba al menos una comorbilidad, destacando la hipertensión arterial (22,4%), seguida de ansiedad/depresión y de obesidad; el 41,1% tenían uno o dos comorbilidades (Tabla 1).

La gran mayoría de pacientes incluidos en el estudio (80,9%) no habían recibido ninguna dosis vacunal COVID antes de la primoinfección. Más de la mitad mostraron síntomas leves-moderados que no requirieron hospitalización, y casi un 40% fueron asintomáticos o paucisintomáticos; solo el 5,8% precisaron ingreso hospitalario (Tabla 1).

**Tabla 1:** Características de la muestra estudiada

Variables	Global	Hombre	Mujer	p (*χ^2^*)
	n (%)	n (%)	n (%)	
Demográficas				
Sexo (mujer)		72 (41,6)	101 (58,4)	
Edad*	47 (16,3)	50.4 (16,9)	44.7 (15,5)	0,02
Clínicas				
Comorbilidades				
A/D	28 (16,2)	10 (13,8)	18 (17,8)	0,48
Obesidad	26 (14,9)	10 (13,8)	16 (15,8)	0,72
HTA	39 (22,5)	21 (29,2)	18 (17,8)	0,07
Diabetes	11 (6,4)	5 (6,9)	6 (5,9)	0,79
EPOC	8 (4,6)	4 (5,5)	4 (3,9)	0,62
Cardiopatía	7 (4)	5 (6,9)	2 (1,9)	0,1
ACV	1 (0,5)	1 (1,3)	0	0,23
Insuficiencia renal	4 (2,3)	2 (2,7)	2 (1,9)	0,73
Cáncer	5 (2,8)	3 (4,1)	2 (1,9)	0,39
Nº comorbilidades*	0,87 (1,0)			
0	85 (49,1)	33 (45,8)	52 (51,5)	0,23
1	43 (24,9)	18 (25)	25 (24,8)	0,23
2	28 (16,2)	11 (15,4)	17 (16,8)	0,39
≥3	17 (9,8)	10 (13,8)	7 (6,9)	0,06
Embarazo	3 (1,7)			
Inmunosupresión	3 (1,7)	2 (66,7)	1 (33,3)	0,37
COVID-19				
Vacunación previa	33 (19,1)	13 (18)	20 (19,8)	0,38
Número de dosis				
1	10 (5,8)	4 (5,5)	6 (5,9)	0,59
2	23 (13,3)	9 (12,5)	14 (13,8)	0,39
Síntomas				
Ausentes/escasos	67 (38,7)	30 (41,7)	37 (36,7)	0,25
Leves	96 (55,5)	35 (48,6)	61 (60,4)	0,06
Graves (hospitalizados)	10 (5,8)	7 (9,7)	3 (2,9)	0,11
Reinfección SARS-CoV-2				
Sí	35 (20,2)	14 (19,4)	21 (20,8)	0,41
No	138 (79,8)	58 (80,6)	80 (79,2)	0,41

*: media (desviación estándar), comparadas con U de Mann-Whitney; A/D: ansiedad/depresión; HTA: hipertensión arterial; EPOC: enfermedad pulmonar obstructiva crónica; ACV: accidente cerebrovascular.

### Persistencia de síntomas COVID

El 32,4% de pacientes (n=56) presentó algún síntoma COVID persistente (durante ≥8semanas) en cualquier momento de los dos años posteriores a la primoinfección por SARS-CoV-2. La mitad de pacientes sintomáticos presentó astenia y más de un tercio presentó anosmia, siendo los síntomas más frecuentes, seguidos por disgeusia, disnea y artralgias (Tabla 2). Las mujeres presentaron síntomas COVID persistente con mayor frecuencia que los hombres (37 de 101= 36,7% frente a 19 de 72= 26,3%; p= 0,07).

La duración media de los síntomas declarados por pacientes con COVID persistente fue globalmente dos meses (DE 5,62); los síntomas más duraderos fueron en general los más frecuentes (Tabla 2). La anosmia y la disgeusia fueron los síntomas de COVID persistente con mayor duración (4-5 meses), seguidos de disnea, artralgias y alteraciones de la concentración (tres meses). Al momento de la recogida de datos, dos años después de la primoinfección por SARS-CoV-2, el 23,1% de pacientes aún presentaban algún síntoma; la frecuencia de artralgias, anosmia y astenia fue ≥15% (Tabla 2). No se observaron diferencias por sexo en la persistencia ni en la duración de ninguno de los síntomas analizados.

**Tabla 2:** Frecuencia y duración de síntomas COVID-19 persistente*

Síntoma	Duración (meses)	Frecuencia n (%)	
	Media (DE)	En cualquier momento del seguimiento	A los 24 meses
Astenia	3,77 (7,52)	30 (53,6)	9 (16,1)
Anosmia	5,08 (10,33)	22 (39,3)	9 (16,1)
Disgeusia	4,45 (10,88)	17 (30,3)	6 (10,7)
Disnea	3,8 (8,49)	16 (28,6)	8 (14,3)
Artralgias	3,65 (8,64)	16 (28,6)	10 (17,8)
Tos	1,34 (5,07)	11 (19,6)	3 (5,3)
Alteraciones de la concentración	3,42 (8,46)	10 (17,8)	8 (14,3)
Apatía	2,48 (7,24)	10 (17,8)	7 (12,5)
Alteraciones del sueño	2,68 (7,32)	10 (17,8)	7 (12,5)
Cefalea	1,07 (4,47)	9 (16,1)	5 (8,9)
Ansiedad	2,08 (6,73)	9 (16,1)	7 (12,5)
Déficit de memoria	2,90 (7,80)	9 (16,1)	7 (12,5)
Palpitaciones	1,54 (5,82)	7 (12,5)	4 (7,1)
Caída de pelo	1,77 (6,30)	7 (12,5)	3 (5,3)
Parestesia	1,73 (6,19)	6 (10,7)	5 (8,9)
Inestabilidad/ mareo	0,46 (3,38)	4 (7,1)	3 (5,3)
Opresión torácica	0,12 (0,78)	2 (3,6)	0
Pérdida de peso	0,25 (1,59)	2 (3,6)	0
Febrícula	0,03 (0,25)	1 (1,7)	0
Escalofríos	0,02 (0,12)	1 (1,7)	0
Pérdida de audición	0	1 (1,7)	0
Diarrea	0,10 (0,78)	1 (1,7)	0
Total	2,03 (5,62)	56 (32,36%)	40 (23,1%)

*: COVID persistente: presencia de al menos un síntoma durante un periodo ≥8 semanas sin otra causa atribuible desde la primoinfección por SARS-COV-2; DE: desviación estándar.

En el análisis multivariante de regresión logística binaria, la variable dependiente *persistencia de síntomas COVID* fue significativamente más probable en pacientes con mayor gravedad de la primoinfección COVID (sintomáticos frente a asintomáticos), con menor edad y que presentaban reinfección por SARS-CoV-2 (Tabla 3).

**Tabla 3:** Variables asociadas a COVID persistente (análisis de regresión logística multivariante)

Variables	Análisis univariante [n (%)]			Análisis multivariante	
	COVID persistente		p (*χ*^2^)	OR (IC95%)	p
	Sí (n=56)	No (n=117)			
Sexo (F)	37 (66,1)	64 (54,7)	0,07	0,97 (0,51 - 1,85)	0,931
Edad*	45,61 (17,9)	50,07 (11,8)	0,056	0,97 (0,95 - 0,98)	0,001
Gravedad			0,001		0,023
Asintomáticos	12 (21,4)	55 (47,0)		1	
Sintomáticos	44 (78,6)	62 (53,0)		2,15 (1,11 - 4,14)	
Reinfección			0,06		0,036
No	39 (69,6)	99 (84,6)		1	
Sí	17 (30,4)	18 (15,4)		1,75 (1,03 - 2,95)	

OR= *odds ratio*; IC: intervalo de confianza; *: media (desviación estándar) comparadas mediante t-Student.

De las 56 personas sintomáticas, el 78,3% presentaron dos o más síntomas persistentes, con una media de 3,37 síntomas por paciente (DE=2,89). El número medio de síntomas fue superior en mujeres aunque de forma no significativa (1,36; DE=2,68 frente a 0,9; DE=1,7; U de Mann Whitney p= 0,18).

El análisis multivariante de regresión lineal (R^2^= 0,37) detectó que los pacientes con depresión, con mayor gravedad de COVID-19 y con reinfección por SARS-CoV-2 mostraban significativamente mayor número de síntomas persistentes (Tabla 4).

**Tabla 4:** Variables asociadas al número de síntomas COVID persistente (regresión lineal múltiple)

Variables	B (IC95%)	p
Depresión (sí/no)	1,53	0,02
	(0,56 - 2,48)	
Gravedad COVID (sintomático/asintomático)	0,94	0,003
	(0,33 - 1,56)	
Reinfección SARS-CoV-2 (sí/no)	0,52	0,051
	(0,01 - 1,03)	

### Calidad de vida relacionada con la salud

Las personas incluidas en el estudio puntuaron su percepción de calidad de vida relacionada con la salud con una media de 83,25 puntos (DE=17,08) y una mediana de 90 puntos sobre 100 (EuroQol-5D); no hubo diferencias por sexo. Las personas sin COVID persistente manifestaron más frecuentemente no tener dolor o malestar (85,5 vs 71,4%; p=0,01) y su puntuación EQ-EVA media fue casi nueve puntos mayor (86,15 vs 77,72; p=0,001). También los pacientes con COVID persistente declararon significativamente más la presencia de *mucha* ansiedad/depresión (12,5 vs 4,2%; p= 0,02) (Tabla 5). No se observaron diferencias por sexo en las subescalas del EuroQol-5D ni en la puntuación media de la escala EQ-EVA (83,35; DE=16,34 en hombres y 83,18; DE=19,67 en mujeres; p=0,94).

**Tabla 5:** Relación entre persistencia de síntomas COVID y calidad de vida

Subescalas EUROQoL-5D	COVID persistente		
	Sí	No	p
	n=56 (32,4%)	n=117 (67,6%)	(*χ*^2^)
Movilidad (problemas para caminar), *n (%)*			
No tengo	54 (96,4)	114 (97,5)	0.77
Algún problema	2 (3,6)	3 (2,5)	0,77
Tengo que estar en la cama	0	0	1
Cuidado personal (problema para lavarme/vestirme), *n (%)*			
No tengo	54 (96,4)	115 (98,3)	0,39
Algunos problemas	2 (3,6)	2 (1,7)	0,39
Soy incapaz	0	0	1
Actividades cotidianas (problemas para realizarlas), *n (%)*			
No tengo	53 (94,6)	114 (97,5)	0,29
Algunos problemas	3 (5,4)	1 (0,8)	0,1
Soy incapaz de realizarlas	0	2 (1,7)	0,45
Dolor/malestar, *n (%)*			
No tengo	40 (71,4)	100 (85,5)	0,01
Moderado	11 (19,6)	13 (11,2)	0,06
Mucho	5 (9,0)	4 (3,3 )	0,12
Ansiedad/depresión, *n (%)*			
No tengo	42 (75,0)	96 (82,1)	0,14
Moderada	7 (12,5)	16 (13,7)	0,41
Mucha	7 (12,5)	5 (4,2)	0,02
Puntuación EVA, *media (DE)*	77,72 (17,34)	86,15 (16,25)	0,001

DE: desviación estándar.

En el análisis multivariante de regresión lineal (R^2^=0,87) se observó que ser mujer aumenta en al menos 5,75 puntos la puntuación del EQ-EVA; a mayor edad y mayor número de dosis vacunal también se encontró mayor puntuación, mientras que un mayor número de comorbilidades se relacionó con una menor puntuación (Tabla 6).

**Tabla 6:** Modelo de regresión lineal para el análisis multivariante de la escala visual analógica del cuestionario EuroQol-5D

Variables	B (IC95%)	p
Sexo (mujer/hombre)	16,496	0,003
	(5,75 - 27,24)	
Edad (años)	1,302	0,0001
	(1,04 - 1,56)	
Comorbilidades (número)	-6,387	0,046
	(-12,65 - -0,12)	
Vacunación (sí/no)	15,546	0,001
	(6,46 - 24,62)	
Gravedad COVID (sintomático/asintomático)	3,365	0,53
	(-7,33 - 14,066)	
Síntomas persistentes (número)*	-0,41	0,67
	(-2,37 - 1,55)	

IC= intervalo de confianza; *: en cualquier momento de los 2 años posteriores a la infección por SARS-CoV-2.

## DISCUSIÓN

Los principales hallazgos de este estudio son una prevalencia de COVID persistente del 32,4%, con síntomas presentes en un 23% de los pacientes hasta dos años después de la infección inicial. Este periodo de seguimiento prolongado es la principal diferencia con otros estudios (77días^28^, 110 días^12^, 7 meses^11^, un año^29^^,^^30^).

La prevalencia de COVID persistente fue menor en el presente estudio (32,36%) que en otros publicados, lo que podría deberse a diferencias en el tipo de pacientes (contexto hospitalario) y al mayor tiempo de seguimiento. En pacientes reclutados desde urgencias se encontró un 50,9% de persistencia de síntomas a 77 días^28^, mientras que pacientes sintomáticos que habían requerido ingreso hospitalario presentaron prevalencias post infección del 68% a los seis meses y del 49% al año^19^.

También se observan diferencias respecto a otros estudios realizados en atención primaria. Un estudio de seguimiento a seis y doce meses considerando una definición similar de síndrome posCOVID-19 (persistencia durante al menos dos meses de síntomas no atribuibles a otro diagnóstico), encontró una prevalencia mucho menor (8,6%) de COVID persistente^29^, que podría explicarse por la exclusión de pacientes mayores de 65 años y por la menor gravedad de sus síntomas (95,7% de infecciones leves y 12,2% asintomáticas). La prevalencia de síntomas persistentes también fue menor (12,39% a los 90 días y 6,29% al año) en otro estudio con pacientes de similar edad y sexo que fueron categorizados en subgrupos por presencia de secuelas, manifestaciones paradójicas o manifestaciones preexistentes^30^, que en el presente estudio no se consideraron de forma separada.

El síntoma más presente en este estudio fue la astenia (fatigabilidad, cansancio intenso que interfiere con la actividad cotidiana), coincidiendo con lo recogido tanto por los Centros para el Control y la Prevención de Enfermedades (CDC)^31^ como por la Organización Mundial de la Salud (OMS)^32^. Al igual que en el metaanálisis de Pinzón y col^33^, encontramos alta frecuencia de síntomas neuropsicológicos. Aunque la duración promedio de los síntomas más frecuentes en esta investigación fue 4-5 meses, algo menor que en el estudio de Rodríguez Onieva y col (alrededor de seis meses)^30^, algunos síntomas como las artralgias, la astenia y la anosmia se extendieron hasta los dos años en más del 15% de pacientes, persistencia prolongada ya descrita por otros estudios^34^^,^^35^.

Hemos encontrado asociación entre mayor gravedad de la infección y mayor presencia de COVID persistente, si bien hubo una baja representación de pacientes hospitalizados (solo 10 pacientes). Se conoce que la COVID persistente puede desarrollarse en pacientes con infecciones agudas leves^35^. El hecho de que el 21,4% de pacientes asintomáticos durante la primoinfección desarrollaran COVID persistente apunta a la falta de evidencia clara sobre su relación con la gravedad de la infección aguda^36^.

Una menor edad de los pacientes se relacionó con COVID persistente, hallazgo concordante con otro estudio^36^ pero discordante con otros en los que la edad mayor de 60 años fue un factor de riesgo para padecer esta entidad^30^. Existen variables que pueden interferir en esta relación, como el estado vacunal (vacunación preferente a mayor edad) o el sesgo de recuerdo (mayor a mayor edad).

Las investigaciones realizadas van acumulando evidencia de que la reinfección por SARS-CoV-2 aumenta el riesgo de COVID persistente si bien también se sugiere que las personas con COVID persistente podrían tener reducidos niveles de anticuerpos frente a la enfermedad, lo que haría más probable la reinfección^37^.

Los pacientes con COVID persistente puntuaron peor en calidad de vida en la subescala de dolor, lo que concuerda con la elevada frecuencia de síntomas como las artralgias, que están presentes en un porcentaje significativo de pacientes a los dos años de la primoinfección. También puntuaron peor en la percepción del estado de salud (EQ-EVA). Rodríguez-Onieva y col^30^ encontraron que a mayor duración de síntomas persistentes, mayor pérdida de capacidad funcional evaluada mediante una escala de 0 a 4.

En este estudio encontramos que el sexo femenino, la edad >65 años, un menor número de comorbilidades y un estado vacunal mas completo fueron las variables asociadas a una mayor puntuación de calidad de vida. Existe evidencia de un mayor riesgo de enfermedad grave y hospitalización en hombres durante la fase aguda de la enfermedad^38^ lo cual podría influir en una peor calidad de vida posterior, en concordancia con nuestros resultados.

De forma similar a lo mostrado en otras investigaciones^39^, observamos que las comorbilidades se relacionaron con mayor número de síntomas posCOVID-19 y con peor calidad de vida; un peor estado funcional previo a la infección puede determinar un aumento sustancial en la necesidad de cuidados posteriores^40^. El contexto temporal de esta investigación corresponde a las etapas iniciales del desarrollo de la campaña de vacunación COVID, en las que la cobertura fue desigual según edad y riesgo, por lo que hemos de valorar con cautela las conclusiones a extraer de los resultados obtenidos respecto al estado vacunal. En 2021 se encuestó a pacientes con COVID persistente, un 26% de los cuales afirmaban haber mejorado con alguna dosis de la vacuna, mientras el 18% manifestaba que había empeorado y el 55% que no había notado diferencias en su estado^41^. Existe alguna evidencia a favor de que la vacuna podría reducir la incidencia de COVID persistente^42^.

Este estudio tiene la fortaleza de incluir pacientes con COVID-19 seguidos en atención primaria y explorar el estado de salud de los pacientes durante un periodo prolongado a dos años desde la infección aguda, duración superior a la de otros estudios. Las principales limitaciones se derivan de la fuente de datos acerca de los síntomas debido a posibles sesgos de memoria de los pacientes y a la dificultad para descartar razonablemente otras causas más probables de los síntomas, ya que se trata en su mayoría de síntomas frecuentes en múltiples patologías. Como potenciales mejoras para investigaciones futuras podría establecerse una cohorte a partir de los pacientes incluidos en este estudio y realizar un seguimiento prospectivo sobre su estado de salud y calidad de vida incluyendo métodos combinados cuantitativos y cualitativos para la recogida de información.

Como conclusiones de esta investigación, el 32% de los pacientes presentaron criterios de COVID persistente en algún momento de los dos años de seguimiento, y un 23% de casos presentaban síntomas a los dos años de la primoinfección, lo que supone un importante porcentaje de pacientes atendidos en las consultas de atención primaria.

Los síntomas más prevalentes fueron el cansancio, la pérdida de gusto y olfato y las artralgias, síntomas frecuentes en nuestras consultas y ante los que se debe incluir el COVID persistente como diagnóstico diferencial. Fue más frecuente en pacientes de menor edad, con primoinfección más grave y con reinfección por SARS-CoV-2. Este conjunto de variables podría considerarse como perfil de riesgo para la aparición de COVID persistente, y ser tenido en cuenta por el personal especialista en Medicina Familiar y Comunitaria que atiende a estas personas. La COVID persistente se asoció con peor calidad de vida, especialmente en relación a la dimensión dolor y a la escala visual de valoración del estado de salud, aunque la calidad de vida fue mejor en pacientes de mayor edad, de sexo femenino, con menos comorbilidades y más dosis vacunales. Esta situación refleja la necesidad de establecer intervenciones multiprofesionales en los centros de salud para mejorar el bienestar de los pacientes afectados.

## Data Availability

Se encuentran disponibles bajo petición al autor de correspondencia.
